# Effectiveness of preconception care interventions in primary care: a systematic review protocol

**DOI:** 10.3399/BJGPO.2021.0191

**Published:** 2022-05-18

**Authors:** Nishadi Nethmini Withanage, Jessica R Botfield, Sonia Srinivasan, Kirsten I Black, Danielle Mazza

**Affiliations:** 1 Department of General Practice, Monash University, Clayton, Australia; 2 Western Health, St Albans, Australia; 3 Discipline of Obstetrics, Gynaecology and Neonatology, Central Clinical School, University of Sydney, Camperdown, NSW, Australia

**Keywords:** preconception care, pre-pregnancy care, pregnancy outcome, primary health care, general practice

## Abstract

**Background:**

Pregnancy outcomes can be adversely affected by a range of modifiable risk factors, including alcohol consumption, smoking, obesity, drug use, and poor nutrition, during the preconception period. Preconception care (PCC) involves interventions that identify and seek to change behavioural, biomedical, and social risks present in reproductive-aged women and men. Primary care is well situated to offer PCC interventions but the effectiveness of these interventions is not clear.

**Aim:**

To evaluate the effectiveness of primary care-based PCC delivered to reproductive-aged women and/or men to improve health knowledge, reduce preconception risk factors, and improve pregnancy outcomes.

**Design & setting:**

A systematic review of primary care-based PCC.

**Method:**

Ovid MEDLINE, Cochrane Central Register of Controlled Trials, Embase, Web of Science, Scopus, and the Cumulative Index to Nursing and Allied Health Literature (CINAHL) databases will be searched for English language studies published between July 1999 and May 2021. For inclusion, the PCC intervention must be provided in a primary care setting and intervention recipients must be reproductive-aged women and/or men. All stages of screening and data extraction will involve a dual review. The Cochrane Risk of Bias 2 (RoB 2) for randomised controlled trials (RCTs) will be used to assess the methodological quality of studies. This protocol adheres to the Preferred Reporting Items for Systematic Reviews and Meta-Analyses Protocols (PRISMA-P) reporting guidelines.

**Conclusion:**

Findings will determine the effectiveness of primary care-based preconception interventions delivered to reproductive-aged women and men on improving health knowledge, reducing risk factors, and improving pregnancy outcomes. Findings will be published in a peer-reviewed journal.

## How this fits in

Primary care providers have an important role in providing patients with PCC by educating patients on reducing potential risk factors that may be impacting their health. However, the effectiveness of PCC interventions delivered in primary care settings on improving pregnancy outcomes is not clear. The systematic review described in this protocol will evaluate the effectiveness of primary care-based PCC delivered to reproductive-aged women and/or men to improve health knowledge, reduce preconception risk factors, and improve pregnancy outcomes. Findings may be used to inform policy and practice for the implementation of PCC in primary care globally.

## Introduction

PCC involves interventions that aim to identify and modify the behavioural, biomedical, and social risks that are present in reproductive-aged women and/or men.^1,2^ These interventions aim to improve pregnancy outcomes and the health of women and infants by managing determinants of poor pregnancy outcomes such as mental health issues, excessive alcohol consumption, smoking, poor nutrition, diabetes, and obesity. PCC interventions include risk screening during the preconception period, preconception counselling, and educating people on the importance of maintaining optimal health during the preconception period.

Previous systematic reviews in this field have demonstrated that PCC interventions provided in community and hospital settings are effective in improving pregnancy outcomes by reducing neural tube defects,^3,4^ pre-eclampsia,^5^ abnormal birth weight,^6^ and preterm birth.^7^ However, there is limited evidence on the effectiveness of primary-care based PCC interventions on improving pregnancy outcomes.^1,8–10^ The previous review investigating the effectiveness of PCC interventions in primary care settings concluded that there was a lack of evidence to determine the effectiveness of PCC on improving pregnancy outcomes.^8^

While preconception issues can be addressed through public health promotion strategies, PCC is ideally placed in primary care settings such as general practices, medical clinics, village and community health centres, and allied health practices as this is usually the first point of healthcare contact for patients.^11^ In these settings, one-on-one consultations can be provided to identify and reduce risk factors (for example, smoking, alcohol consumption, and obesity) and educate women and their partners.^12,13^ The potential role of primary care in delivering PCC has been recognised and primary care practitioners have acknowledged that PCC can enhance knowledge of risk factors affecting the preconception period and may improve pregnancy outcomes.^14–18^ While PCC in primary care can be provided by GPs, nurses,^19^ midwives,^20^and non-healthcare professionals,^20–29^ it is often not routine practice in primary care settings or may be considered a low priority.^19,20^

Furthermore, most primary care-based PCC interventions have primarily focused on women.^8,9,13,30,31^ Lifestyle factors such as alcohol consumption and smoking may cause deoxyribonucleic acid (DNA) damage to the sperm, however, which may result in birth defects.^32^ Inclusion of both women and men or partners in PCC may have additional benefits, including positive pregnancy and neonatal outcomes.^33^ For example, men who receive preconception information may be more likely to reduce alcohol consumption, reduce smoking, and consume a healthy diet, which can contribute to optimising paternal health, maternal health, pregnancy, and neonatal outcomes.^32^

A number of studies evaluating the effectiveness of PCC interventions in primary care settings have been published since the previous review. Therefore, a systematic review will be conducted to evaluate the evidence on the effectiveness of primary care-based PCC interventions delivered to reproductive-aged women and/or men to improve health knowledge, reduce preconception risk factors, and improve pregnancy outcomes. This will build on a previous review published in 2016 that focused on women and included RCTs published between July 1999 and July 2015.^8^ The findings of this review may be used to inform policy and practice, and may support the widespread implementation of PCC in primary care globally.

## Method

The preferred reporting process outlined within the PRISMA-P will be adhered to,^34^ and this systematic review has been registered with PROSPERO. A collaborative approach was taken by the authors to develop the objectives, search strategy, and the methodology, guided by the Population, Intervention, Comparison, Outcome (PICO) format.

### Study design

A pilot search was conducted using Google Scholar and the Cochrane Library to identify similar reviews, background literature, and to estimate the volume of published literature on this topic. A number of studies conducted since the last systematic review in 2016 were found.^8^ Some of these studies involved non-healthcare professionals such as the researcher delivering the intervention.^28,35^ The pilot search for this review also showed that meta-analysis cannot be undertaken owing to the heterogeneity of the outcomes investigated across the different studies. The systematic review will follow the four major steps for conducting narrative synthesis in reviews of intervention effectiveness: (1) developing a theory of how and why the intervention works, and for whom; (2) developing a preliminary synthesis of the included studies; (3) exploring relationships in the data within and between studies; and (4) assessing the robustness of the synthesis.^36^ Only RCTs that are focused on PCC will be included, as RCTs are the reference standard for studying causal relationships between interventions and outcomes.^37^ Observational studies will be excluded owing to potential bias associated with these study designs. Reference lists of included articles will be manually screened for additional studies that meet the eligibility criteria. The pilot search demonstrated a low number of studies likely to proceed to full-text screening, therefore manually searching reference lists of included studies is a feasible method of increasing data to inform this review. Grey literature will not be included since this study has been limited to include only RCTs.

### Study setting

In this review, studies identified as being conducted in the primary care setting will include family or general practices, community an village health centres or services, community or outpatient clinics, and ambulatory care services.^8^ Studies will be excluded if the interventions are based in emergency departments, hospital inpatient settings, or other non-primary care settings.

### Population

Studies investigating PCC in women and men of reproductive age (18–45 years) will be included. Interventions delivered by any provider will be eligible; for example, physicians, physician assistants, community/village health workers, nurses/nurse practitioners, midwives, or non-healthcare professionals including researcher-directed PCC in primary care settings.

### Interventions and comparisons

Studies evaluating PCC interventions in primary care settings will be included if the intervention is conducted before conception. Based on previous systematic reviews,^7,8,30,38,39^ interventions may include but are not limited to providing the following: advice, immunisations, education, counselling, biomedical health interventions, reproductive planning, and sexual health risk screening during the preconception period. Intervention groups will be compared with ‘no pre-conception care’ or ‘usual care’.

### Outcomes

The pilot search and previous reviews on this topic^8^ demonstrated that the nature of interventions and outcome measures varied between individual studies. Therefore, primary outcomes will include (but are not limited to) knowledge of factors that affect health during the preconception period, and pregnancy outcomes including the following: maternal morbidity, prematurity and birth weight, fetal or neonatal mortality and morbidity, and fetal abnormalities. Knowledge and awareness of risk factors will be measured via information gathered from knowledge tests and surveys, and interviews with participants. Secondary outcomes will include reduction in modifiable risk factors including but not limited to the following: weight, drug use, alcohol consumption, and smoking.

### Search strategy

A uniform strategy will be developed in consultation with a Monash University search specialist librarian (Supplementary Table S1). Search terms will focus on the population (reproductive-aged human women and men; 18–45 years) and intervention (**Table 1**). The search strategy will be developed using Ovid MEDLINE(R) and Epub Ahead of Print, In-Process, In-Data-Review & Other Non-Indexed Citations, Daily and Versions(R) 1946–1 June 2021, ensuring a combination of relevant medical subject headings and keyword terms. The search will then be adapted to Cochrane Central Register of Controlled Trials, Embase, Scopus, CINAHL, and Web of Science by adjusting the subject headings to other thesauruses and keyword truncation, and phrase searching where necessary. The results from the database searches will be saved in Covidence and duplicates will be removed.

**Table 1.** PICO criteria

Population terms	Teen* or adolescen* or youth or men or man or female or male or woman or women or reproductive age or child bearing age or childbearing age
Intervention terms	preconcept* or pre concept* or interconcept* or prepregnan* or pre pregnan* or pregnanc* plan* or plan* pregnanc* adj8 health program* or health education or health promot* or advic* or advis* or intervention* or care or assess* or risk or counsel* or screen* or folic acid supplement* or folate supplement*
Comparison	No preconception care or usual care
Outcomes	Primary outcomes will include but not limited to: knowledge of factors that affect health during the preconception period, and pregnancy outcomes including: maternal morbidity, prematurity and birth weight, fetal or neonatal mortality and morbidity, and fetal abnormalities.Secondary outcomes will include reduction in modifiable risk factors including but not limited to: weight, drug use, alcohol consumption, and smoking.(No specific key terms for outcomes were included when developing the search strategy owing to the heterogeneity of the outcomes measured across individual studies.)

PICO = Population, Intervention, Comparison, Outcome.

Articles will be included if the study: (a) reports on the effectiveness of PCC in primary care; (b) includes reproductive-aged men and/or women (18–45 years); (c) is an RCT; (d) is written in English; and (e) is published in a peer-reviewed journal between July 1999 and May 2021. The previous systematic review in this field included RCTs from July 1999–July 2015.^8^ To capture new RCTs over the past 6 years and to include RCTs involving men that have been conducted over the past two decades, the timeframe of July 1999–May 2021 was chosen.^4^ The start date was selected following the end of the search of an earlier review by Korenbrot *et al*.^4^ Reference lists of included studies and previous reviews will be manually screened for additional studies meeting inclusion. No geographical limits will be applied. Articles will be excluded if the study: (a) is not conducted in primary care; or (b) uses an observational study design; or (c) includes pregnant women; or (d) focuses on improving fertility. Two reviewers (NW and SS) will independently screen articles for eligibility. Any discrepancies will be discussed with a third reviewer (JB) to reach consensus.

### Data extraction and synthesis

A data extraction form will be created, utilising previous reviews,^8,9,31^ which will include country, study design, setting, population, provider, details of interventions, comparator, and outcomes. A meta-analysis will not be performed owing to the heterogeneity of the outcomes measured across the studies.

NW and SS will independently evaluate included RCTs for the risk of bias using the Cochrane RoB 2 tool for RCTs^40^ with six assessment criteria (sequence generation, allocation concealment, blinding of participants and personnel, blinding of outcome assessors, incomplete outcome data, and selective reporting bias). Studies will be classified as low risk of bias (high quality if four or more criteria with low risk of bias and another two must not be incomplete outcome data or reporting bias), unclear risk of bias (medium quality if at least one criteria had an unclear risk of bias with no incomplete outcome data or reporting bias), or high risk of bias (low quality if at least four criteria had high risk of bias) as classified in previous systematic reviews.^41^ Guidelines for risk of bias will be followed to report risk of bias within the systematic review. If data presentation is problematic, unclear, missing, or presented in an unextractable form, the respective authors will be contacted to minimise the risk of bias and to avoid the inappropriate description of study results.

## Discussion

This systematic review will provide a synthesis of evidence from peer-reviewed studies about the effectiveness of the PCC interventions in primary care published over the past two decades. This will be the first systematic review of primary-care based PCC that includes men and also the first to consider the role of the provider (that is, healthcare professionals and non-healthcare professionals) in the delivery of primary-care based PCC. Five databases will be systematically searched for literature, but it is possible that relevant articles may be missed owing to the search strategies employed. Reference lists of included articles will be reviewed to mitigate this issue. Two additional limitations will be not accounting for publication bias and restricting the eligibility criteria to RCTs only. Despite these limitations, it is anticipated that this systematic review will make an important contribution to the evidence regarding primary care-based PCC for several reasons. First, this review will aim to evaluate the recent evidence on the effectiveness of primary care-based PCC interventions. Second, this is the first systematic review to investigate the importance of primary care-based PCC interventions in both reproductive-aged women and men, and will aim to address how the provision of PCC to reproductive-aged women and/or men may improve health knowledge, reduce preconception risk factors, and improve pregnancy outcomes. Third, to the authors' knowledge, this will be the first review to consider the role of the provider in the delivery of PCC in primary care. Findings may support inclusion of a range of primary healthcare professionals, such as nurses and midwives and other non-healthcare professionals, to broaden access to PCC for reproductive-aged women and/or men. Finally, the results from the review may support the widespread implementation of PCC in primary care globally and contribute to optimising maternal and infant health.

The findings of this study will be presented at national and international scientific meetings and conferences, and will be published in a peer-reviewed journal.

## Trial registration number

Registered with PROSPERO: CRD42021235499.
